# mBeRFP, an Improved Large Stokes Shift Red Fluorescent Protein

**DOI:** 10.1371/journal.pone.0064849

**Published:** 2013-06-20

**Authors:** Jie Yang, Liang Wang, Fei Yang, Haiming Luo, Lingling Xu, Jinling Lu, Shaoqun Zeng, Zhihong Zhang

**Affiliations:** 1 Britton Chance Center for Biomedical Photonics, Wuhan National Laboratory for Optoelectronics, Huazhong University of Science and Technology, Wuhan, Hubei, China; 2 MoE Key Laboratory for Biomedical Photonics, Department of Biomedical Engineering, Huazhong University of Science and Technology, Wuhan, Hubei, China; Cardiff University, United Kingdom

## Abstract

Herein, we describe the generation of a monomeric large Stokes shift (LSS) red fluorescent protein, mBeRFP, with excitation and emission peaks at 446 and 615 nm, respectively. Compared with two previously reported LSS-RFPs (mKeima and LSS-mKate2), mBeRFP is approximately three times brighter. In addition, mBeRFP is characterized by improved photostability, rapid maturation, an extended lifetime, and a monomeric nature. Additionally, mBeRFP can be paired with the Alexa 647 dye as a FRET donor to detect caspase 3 activity. This FRET pair has an extremely dynamic range and a large Förster radius (approximately 6.5 nm). To demonstrate the applicability of mBeRFP for imaging in living cells, we performed dual-color imaging of mBeRFP and CFP simultaneously excited by a single excitation source, and we demonstrated that these fluorescent proteins allow the clear visualization of the dynamics of Bax during cancer cell apoptosis. Thus, mBeRFP appears to be particularly useful for cellular imaging applications.

## Introduction

Many types of fluorescent proteins with various emission wavelengths cloned from marine organisms have been widely used as probes for the non-invasive imaging of proteins, organelles, and cells in real time [1]–[5]. Fluorescence proteins with a large Stokes shift (LSS), i.e., a large gap between the excitation and emission peaks, have many advantages for imaging. LSS red fluorescent proteins (RFPs, Stokes shift >150 nm) have attracted much attention in recent years [6], [7]. These RFPs have several advantages as indicators for molecular tracing in living cells. First, because LSS-RFPs can be excited by blue light with wavelengths between 420 and 450 nm, wavelengths that can also excite CFP or GFP, LSS-RFPs can be used along with CFP or GFP to simultaneously trace the localization of multiple proteins in living cells using only blue light excitation [6], [7]. Second, because LSS-RFPs have a large gap between the excitation and emission peaks, there is little spectral contamination from direct excitation when these proteins are used as FRET donors, similar to mSapphire, mAmertrin, or LSSmOrange, which are large Stokes shift green, yellow, and orange fluorescent proteins, respectively [8]–[10]. Third, LSS-RFPs are also a better choice for the visualization of cell motility, the localization of proteins and changes in gene activity in intact tissues and living organisms when using two-photon (2P) laser scanning microscopy [7], [11]. Recently, three LSS-RFPs, mKeima [6], LSS-mKate1, and LSS-mKate2 [7], which are suitable for molecular imaging in cells have been reported. However, all of these LSS-RFPs have limited brightness, slow maturation times, and low photostability to some extent, thus limiting their use in cellular imaging applications.

In the current study, we performed site-directed mutagenesis on far-red mKate [12] to develop the brightest LSS-RFP obtained to date, which we have named mBeRFP (monomeric Blue light-excited RFP). mBeRFP is a monomer and has improved brightness and photostability as well as a faster maturation time compared with mKeima and LSS-mKate2. The LSS facilitates channel separation and the use of FPs in FRET-based applications and other applications that demand multi-color imaging. For FRET applications, mBeRFP can be paired with the FRET acceptor Alexa 647, which ensures high FRET efficiency. To demonstrate the possible multi-color imaging applications, simultaneous dual-color imaging using mBeRFP and mCerulean, an improved variant of CFP [13], with excitation using a single laser light source at 458 nm was used to track the distribution of the Bax protein and assess the form of the mitochondria during cell apoptosis.

## Materials and Methods

### Site-directed mutagenesis

Site-directed mutagenesis was performed using the megaprimer PCR method with pRSETb-mKate as the template. The sequences of the mutagenic oligonucleotide primers are listed in Table S1. The PCR products were digested with *EcoR*I and *Xho*I and cloned into a modified pRSETb vector, which was subsequently transformed into DH5α competent cells. The cells were cultured on LB plates containing 50 µg/ml ampicillin. After an overnight incubation at 37°C, the overall brightness of individual colonies was assessed using a fluorescence stereomicroscope (Leica MZ FL III; Wetzlar, Germany) equipped with the appropriate filters (excitation 445/20 nm, emission 620/60 nm). The plasmids were extracted using miniprep kits, and the brightest colony was selected for sequencing analysis.

### Plasmid construction and gene transfection

We fused the tubulin gene to the C-terminus of the mBeRFP gene in the pCDNA3.0 plasmid. The human Bax gene was amplified with the primers Bax-F and Bax-R (Table S1) and subsequently cloned into the pmCerulean-C1 vector. A mitochondrial localization sequence was linked to the mBeRFP gene to construct mt-mBeRFP. The plasmids containing mCerulean-Bax and mt-mBeRFP were co-transfected into HeLa cells using Lipofectamine™ 2000.

### Protein expression, purification, and gel filtration

LSS-mKate2, mKeima, and mBeRFP were subcloned into the expression vector pRSETb and subsequently expressed in *E. coli* BL21(DE3) cells. Expression was induced by the addition of isopropyl-thio-β-D-galactopyranoside (IPTG) to a final concentration of 1 mM, and the cultures were grown at 28°C overnight. The proteins were purified using a Ni-NTA column according to the manufacturer's protocol. All purified recombinant proteins were dialyzed against PBS (pH = 7.5) overnight at 4°C. Gel filtration was performed using a 1×90 cm Econo-column (Bio-Rad) packed with Sephacryl-S200 (Amersham) and equilibrated with 25 mM Tris-HCl, pH 7.5, and 150 mM NaCl.

### pH titrations and maturation kinetics

pH titrations were performed using a series of buffers prepared with pH values ranging from 4.0 to 10.0 as previously described [14]. For each pH, 10 µl of purified protein in PBS was diluted by the addition of 100 µl of the corresponding buffer solution, and the fluorescence was measured using an ASCO FP-6500 spectrofluorometer (Japan). To study the maturation of the recombinant proteins, we followed detailed protocols that have been described previously [15]. The maturation time courses were analyzed using a 96-well plate reader (TECAN, Switzerland).

### Spectral analysis

Fluorescence emission spectra from 460 to 650 nm were recorded upon excitation at 430 nm with a bandwidth of 5 nm. All fluorescence spectra were measured using an ASCO FP-6500 spectrofluorometer (Japan). Absorption spectra were acquired using a Perkin Elmer Lambda 35 UV/Visible spectrophotometer. Extinction coefficients were measured using the “alkali-denatured” method [16]. Briefly, mBeRFP and other variants were alkali-denatured with an equal volume of 2 M NaOH. Under these conditions, mBeRFP chromophores were converted to GFP-like chromophores with an extinction coefficient of 44,000 M^−1^cm^−1^ at 446 nm. The molar extinction coefficients were calculated based on the absorbance spectra of the denatured and native FPs. For quantum yield determination, the fluorescence of the mutants was compared with that of the equally absorbing mKeima (the quantum yield for mKeima was measured at 0.24 [6]).

### Fluorescence lifetime imaging

The multi-wavelength TCSPC FLIM system was based on an Olympus FV1000 scan head and a multi-dimensional TCSPC module (SPC-830, Becker and Hickl) with a Ti:sapphire laser (Mai Tai BB, Newport). To perform the FLIM-FRET measurements, a multi-wavelength detector was used (PML-16, Becker and Hickl; a 16-channel detector with a polychromator for spectrally splitting the detected fluorescence signal). The detector was connected to one confocal detection port of the FV1000 scan head using a fiber bundle. The average lifetimes were calculated based on the data obtained.

### Cell culture, induction of apoptosis and confocal imaging

HeLa cells were grown on glass coverslips in Dulbecco's modified Eagle's medium (DMEM, GIBCO) supplemented with 100 units/ml penicillin G sodium, 100 µg/ml streptomycin sulfate and 10% fetal bovine serum and were incubated at 37°C in a humidified atmosphere containing 5% CO_2_. Apoptosis was induced by exposing the cells to 30 µg/ml cisplatin (Sigma). The cells were imaged using an Olympus FV1000 confocal microscope. An argon laser (458 nm) was used to excite mCerulean and mBeRFP, and the fluorescence emission signals for these proteins were monitored using channel 1 (465–490 nm) and channel 2 (590–640 nm), respectively.

## Results

### Development of a bright LSS-RFP

**Figure 1:**
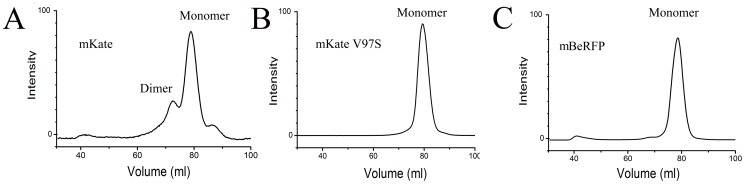
Characterization of the purified variants. (A) The normalized excitation (solid line) and emission (dashed line) spectra of mBeRFP. (B) Normalized curve of the maturation times of mBeRFP (solid line), mLSS-mKate2 (dashed line), and mKeima (dotted line). (C) Absorption spectra at various pH values (4–10). (D) Photobleaching times of mBeRFP (solid line), mLSS-mKate2 (dashed line), and mKeima (dotted line).

To develop LSS-RFPs with good physical and chemical properties, we chose mKate as a precursor because it is a bright far-red fluorescent protein. However, according to previous reports, mKate exists primarily in a monomeric form with a small population of weak dimers [17]. To further verify this distribution of monomers and dimers, we performed gel filtration and found that the majority of mKate proteins appeared to be monomers, though there was concomitant weak dimer formation (Figure 1A). The crystal structure of mKate [18] (Figure S2) suggested that two hydrophobic amino acids (Val97 and Val101) may be the key sites involved in the formation of the weak dimers. When Val97 of mKate was mutated to a serine, the results of the gel filtration analysis indicated that the mKate-V97S variant was present only in a monomeric form (Figure 1B). To further generate the new LSS variant, we used site-specific random mutagenesis to modify the amino acids surrounding the chromophore of mKate-V97S. Using degenerate primers, we first mutated position 162 in the mKate-V97S variant (Trp, Tyr, Asp, His, Phe, Ala). Among the mutagenesis clones, we found a variant, mKate-V97S/S162D, that exhibited altered spectral properties and was capable of being excited by blue light (420–450 nm). To screen for brighter variants, we further mutated mKate V97S/S162D using saturation mutagenesis at position 178 combined with random mutagenesis. The brightest variant of mKate that we obtained, V97S/S162D/D163Y/L178A/Y214S, was named mBeRFP (monomeric Blue light-excited Red Fluorescent Protein).

### Characterization of the purified variants

**Figure 2:**
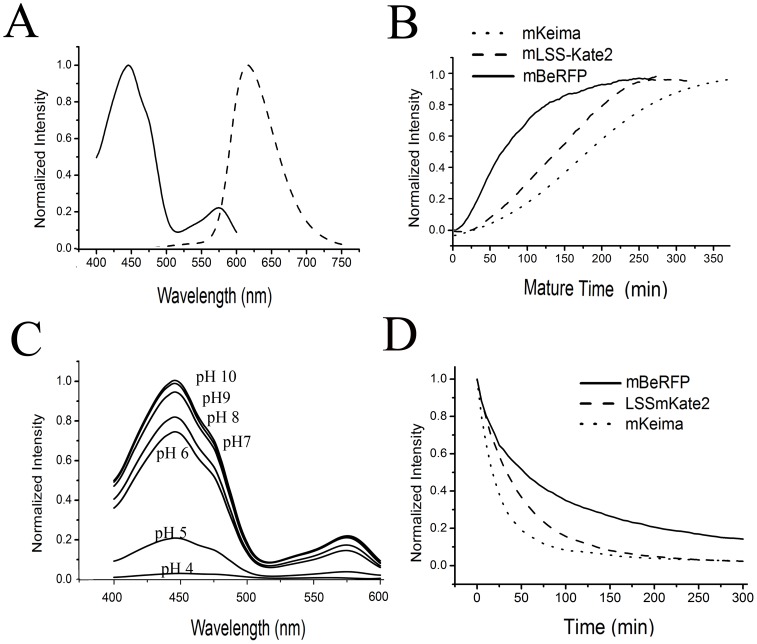
Gel filtration spectra of mKate (A), mKate V97S (B), and mBeRFP (C).

We characterized the purified mBeRFP protein and compared its properties with those of mKeima and LSS-mKate2. The main characteristics of mBeRFP are summarized in Table 1. mBeRFP exhibited excitation and emission peaks at 446 and 611 nm, respectively (Figure 2A). Similar to mKeima and LSS-mKate2, mBeRFP can be excited by blue light (excitation wavelength, λex = 450 nm) owing to a protonated ground state, and it has a red emission peak at 611 nm (emission wavelength, λem = 611 nm). In addition to its major excitation peak at 446 nm, mBeRFP has a minor excitation peak at 580 nm.

**Table 1:** Characteristics of the mBeRFP variant in comparison with mKeima and LSS-mKate2.

Protein	Ex(nm)	Em(nm)	Emol [M^−1^ cm^−1^]	QY	Relative brightness	Photo-stability(min)	pKa	Maturation at 37°C (min)	Lifetime(ns)
mBeRFP	446	611	65,000 (446 nm)&	0.27#	3.2	60	5.6	60	2.0
LSS-mKate2	453	605	37,000 (450 nm)	0.13#	1	32	2.7	130	1.4
			26,000 (453 nm)*	0.17*					
mKeima	440	620	27,300 (438 nm)	0.24*	1.2	21	6.5	180	1.8
			14,400 (440 nm)*						

The QYs of mBeRFP (0.27) and LSS-mKate2 (0.13) were obtained using mKeima (0.24) as a reference.

* Values obtained from previously published data (Kogure et al, 2006; Piatkevich et al, 2010).

# QYs of mBeRFP and LSS-mKate2 were obtained using mKeima (0.24) as a reference.

& Concentration of the chromophore determined by the alkaline denaturation method. [14], [15].

LSS-mKate1 is not listed here for comparison because it is much dimmer than LSS-mKate1 (Piatkevich et al, 2010).

To assay the brightness of mBeRFP, the molar extinction coefficient of mBeRFP was measured using the alkaline denaturation assay [4] and was determined to be 65,000 M^−1^cm^−1^ (446 nm). The QY of mBeRFP (0.27) was obtained using mKeima (0.24) as a reference. According to these measurements, the relative molecular brightness of mBeRFP is approximately three times brighter than those of mKeima and LSS-mKate2. To confirm that mBeRFP was the brightest of these fluorescent proteins, we used *E. coli* BL21(DE3) cells to express these proteins under the same conditions. As expected, the mBeRFP clone of *E. coli* exhibited the brightest fluorescence on the culture plate (Figure S3). Moreover, our data indicate that the maturation half-time of mBeRFP is 60 min at 37°C (versus 180 min for mKeima and 130 min for LSS-mKate) (Figure 2B and Table 1). The pKa value of mBeRFP (5.6) indicates that this protein is relatively stable in an acidic environment (Figure 2C and Table 1).

Fluorescent proteins used in long-term imaging studies need to exhibit a high level of photostability. To determine the photostability of mBeRFP in comparison with several established fluorescent proteins, we performed photobleaching experiments in *E. coli* cells. The photobleaching experiments were conducted using widefield fluorescence microscopy with metal halide illumination and commercial filter sets. The results showed that mBeRFP is more photostable than other LSS proteins, including LSS-mKate2 and mKeima (Figure 2D).

We used a gel-filtration assay to confirm that mBeRFP is a true monomer. The results of this assay showed that mBeRFP exists in a stable monomeric form (Figure 1C). mBeRFP contains a mutation at position 97 (V97S) and exhibits the same monomeric characteristics as mKate-V97S.

### mBeRFP paired with Alexa 647 as a FRET donor/acceptor pair

**Figure 3:**
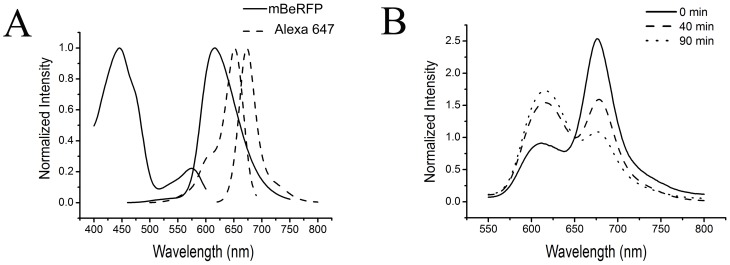
mBeRFP was paired with Alexa 647 to form a FRET donor/acceptor pair. (A) The fluorescence spectra of mBeRFP (solid line) and Alexa 647 (dashed line). (B) The mBeRFP-DEVD-SNAP 647 protein was digested by caspase 3 *in vitro* at 37°C. The spectral sign was recorded at the 0, 40, and 90 min time points.

To demonstrate the use of mBeRFP in a FRET experiment, we developed a new FRET pair, with mBeRFP and Alexa 647 functioning as the donor and acceptor, respectively. Alexa 647 is a far-red dye with a high level of brightness (ε = 239,000 M^−1^cm^−1^, Φ = 0.2) (Maurel et al. 2008). Additionally, Alexa 647 cannot be directly excited by a 458-nm light source (Fig. 3A). According to the calculation of the Förster radius [19], [20], mBeRFP and Alexa 647 have a FRET radius of approximately 6.5 nm. To link mBeRFP with the small chemical dye Alexa 647, we used the SNAP-tag technique. The SNAP tag was derived from the O6-guanine nucleotide alkyltransferase and covalently reacts with benzylguanine-Alexa 647 [21].

We designed a genetically encoded caspase 3 FRET indicator using mBeRFP and SNAP-Alexa 647 linked by the caspase 3 substrate sequence DEVE. When purified mBeRFP-DEVE-SNAP covalently reacted with benzylguanine-Alexa 647, the spectral results showed that the fluorescence was effectively transferred from mBeRFP to the SNAP-Alexa 647 moiety. After cleavage by caspase 3, the FRET signal gradually disappeared (Figure 3B). The enzyme-cleavage reaction caused a 5-fold change in the FRET signal. Because the Alexa 647 dye cannot cross the membrane of living cells, the mBeRFP-Alexa 647 sensor can be used only in *in vitro* assays or assays of cell surface proteins. Because mBeRFP is brighter than LSS-mKate2 and mKeima, it is the best FRET donor for use with the acceptor Alexa 647.

### The properties of mBeRFP in living cells

**Figure 4:**
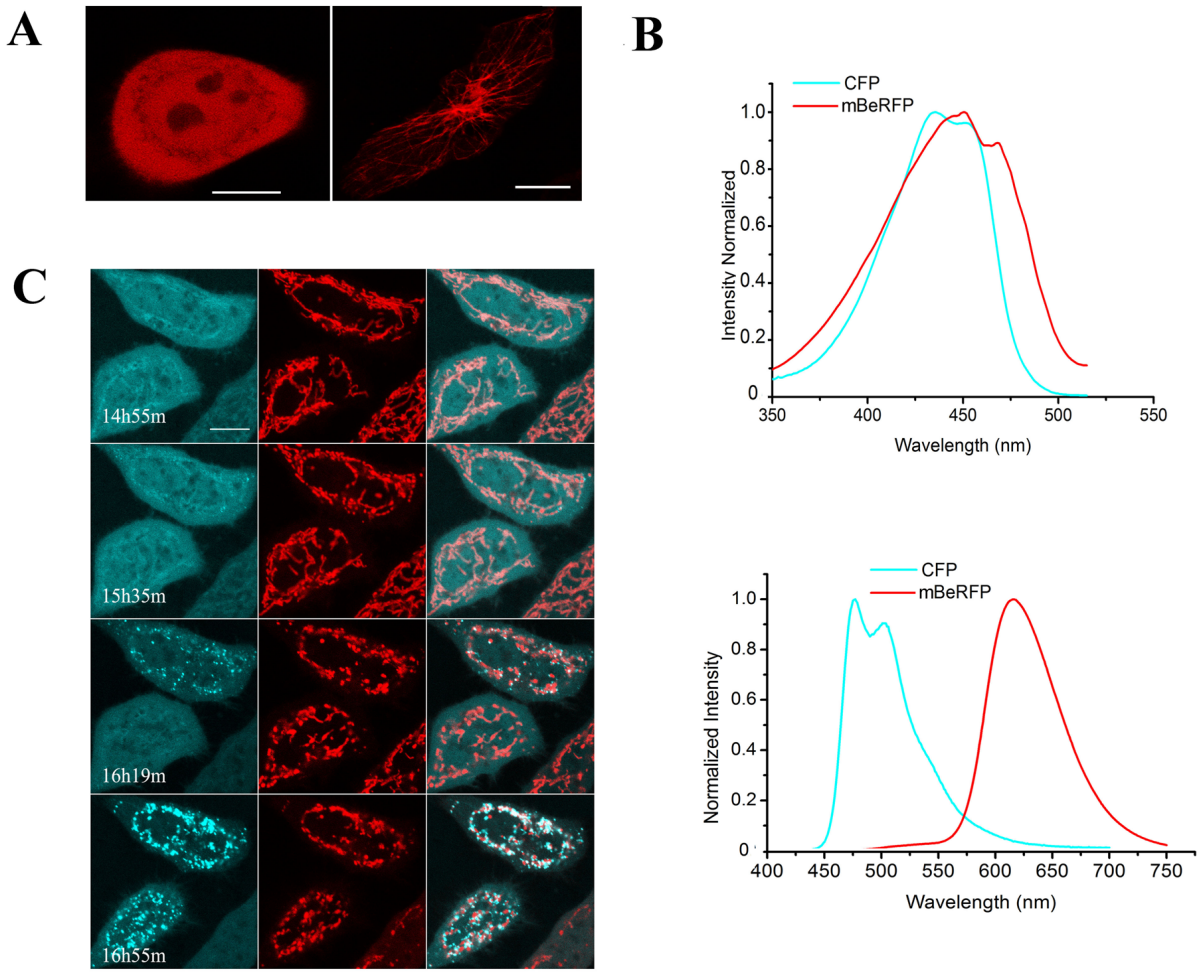
Use of mBeRFP in the fluorescent imaging of living cells. (A) Left panel: pcDNA3.0-mBeRFP expressed in a HeLa cell. Right panel: The chimeric protein mBeRFP-α-tubulin (human) expressed in a B16 cell (mouse melanoma cell line). (B) Upper panel: excitation spectra of CFP (cyan line) and mBeRFP (red line). Lower panel: emission spectra of CFP (cyan line) and mBeRFP (red line). (C) The chimeric proteins mCerulean-Bax and mt-mBeRFP were co-expressed in HeLa cells. The cells were exposed to 30 µg/mL cisplatin to induce apoptosis. Dual fluorescent signals were recorded after the cells were treated (scale bar = 10 µm).

To characterize mBeRFP in living cells, we transiently transfected HeLa cells with mBeRFP without any target gene and imaged the cells using a confocal microscope (Figure 4A, left panel). The fluorescent signal was evenly distributed throughout the nucleus and cytosol of living HeLa cells, without any nonspecific localization or aggregation. We then fused mBeRFP with human α-tubulin. The fusion constructs exhibited a localization pattern that was similar to that of the endogenous protein, indicating that mBeRFP also exhibits monomeric behavior in mouse melanoma B16 cells (Figure 4A right panel). The excitation spectrum of mBeRFP extensively overlaps with that of mCerulean (Figure 4B upper panel); however, their emission spectra are quite separate (Figure 4B lower panel). mCerulean and mBeRFP can be simultaneously excited by 458-nm laser light in confocal microscopy experiments, and their emission signals can be easily detected by different photomultiplier tubes. Therefore, mBeRFP and mCerulean can be simultaneously imaged in a single cell for long-term imaging experiments without the need to reset the imaging software parameters during signal acquisition. To dynamically image two molecules, we fused mBeRFP with a mitochondrial localization sequence and mCerulean (a CFP variant) with Bax, which is a proapoptotic member of the Bcl-2 family and translocates to discrete foci on mitochondria during the initial stages of apoptosis [22], [23]. After the cells were co-transformed with the two constructs and exposed to 30 µg/mL cisplatin for 8 h, we observed four discrete Bax distribution patterns (Figure 4C). First, Bax was primarily distributed evenly throughout the cytosol, with some mitochondrial staining. Next, mCerulean-Bax began to translocate to the mitochondria and exhibited an uneven distribution at the mitochondria. Subsequently, mCerulean-Bax began to concentrate at a few small, isolated fluorescent spots. Finally, the small Bax complexes gradually expanded to form large clusters by recruiting additional Bax molecules. During the entire process, mBeRFP and mCerulean exhibited bright, clear fluorescent signals.

## Discussion

The primary aim of this study was to develop a bright LSS-RFP. Our molecular evolution method resulted in the development of the mBeRFP variant. There are five amino acid mutations in mBeRFP that distinguish it from the parental mKate protein (Figure S1). Among these mutations, S162D and L178A are within the barrel fold, and V97S, D163Y, and Y214S are external to the fold. According to the crystal structure of mKate, the hydroxyl group of Tyr68 in the chromophore is closest to positions Ser147 and Glu162. We hypothesize that this tyrosine hydroxyl in the chromophore forms hydrogen bonds with Glu162 in mBeRFP. The pKa of the carboxyl group of Glu162 is lower than that of the Tyr68 side chain hydroxyl, which suggests that the chromophore can be stabilized in a neutral form. The side chain of Glu162 can form a hydrogen bond with the chromophore, thus changing the environmental pH. Similar to what was observed for mKeima and LSS-mKate, this structure may be primarily responsible for the LSS due to an excited-state proton transfer (ESPT) from the tyrosine hydroxyl in the chromophore to the side chain of Glu162. Similar to mKeima, mBeRFP has two excitation peaks: a main peak at 446 nm and a minor peak at 576 nm. This result suggests that the chromophore simultaneously exists in neutral and deprotonated states. Due to its minor excitation peak at 576 nm, mBeRFP is not suitable for multi-color imaging with RFP; however, the exceptional properties of mBeRFP make it a better tool than the previously employed LSS-RFPs.

## Supporting Information

Figure S1 **Sequence alignment of mBeRFP and its predecessors, mKate and LSS-mKate2.** All identical residues are marked blue, and mutated residues are shown in white.
(TIF)

Figure S2 **Crystal structure of mKate.** Two closely situated hydrophobic amino acids (Val97 and Val101) in different monomers may be the key sites involved in the formation of the weak dimers.
(TIF)

Figure S3 **Brightness of mBeRFP, LSS-mKate2, and mKeima expressed in** ***E. coli*** **BL21(DE3) cells.** The pRSET-mBeRFP, pRSET-LSS-mKate2, and pRSET-mKeima plasmids were transformed into *E. coli* BL21(DE3) cells, which were then incubated at 37°C for 24 hours. The images were acquired using a homemade imaging system with an excitation filter at 440–460 nm and an emission filter at 600–640 nm.
(TIF)

Table S1 **Oligonucleotide primers used in this report (mutated positions are underlined, and N indicates A, T, G, or C).**
(DOCX)
