# Supplementary figures and images for: mBeRFP, an Improved Large Stokes Shift Red Fluorescent Protein
Source: PLoS One. 2013 Jun 20;8(6):e64849. doi: 10.1371/journal.pone.0064849 (PMC3688735; doi:10.1371/journal.pone.0064849)

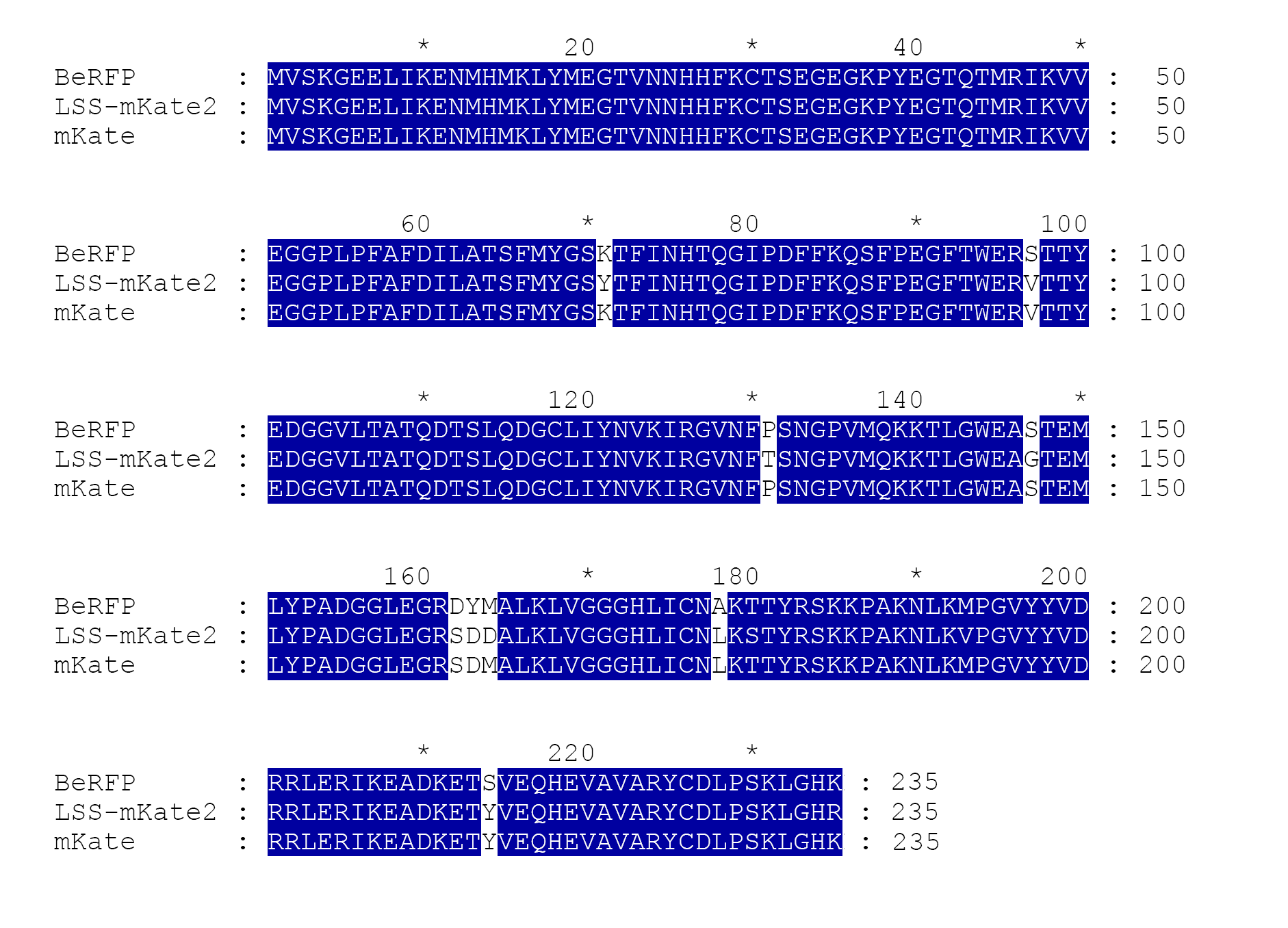

Supplement: Figure S1 — Sequence alignment of mBeRFP and its predecessors, mKate and LSS-mKate2. All identical residues are marked blue, and mutated residues are shown in white. (TIF) [file pone.0064849.s001.tif]

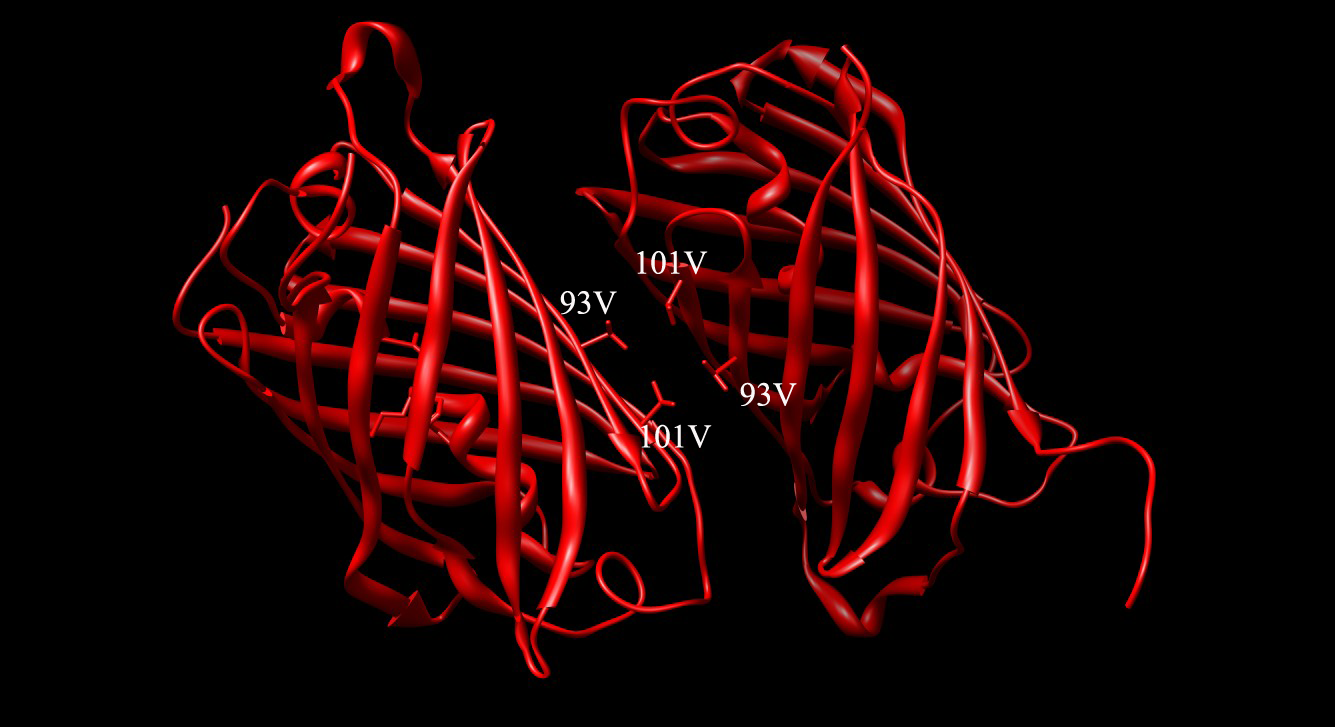

Supplement: Figure S2 — Crystal structure of mKate. Two closely situated hydrophobic amino acids (Val97 and Val101) in different monomers may be the key sites involved in the formation of the weak dimers. (TIF) [file pone.0064849.s002.tif]

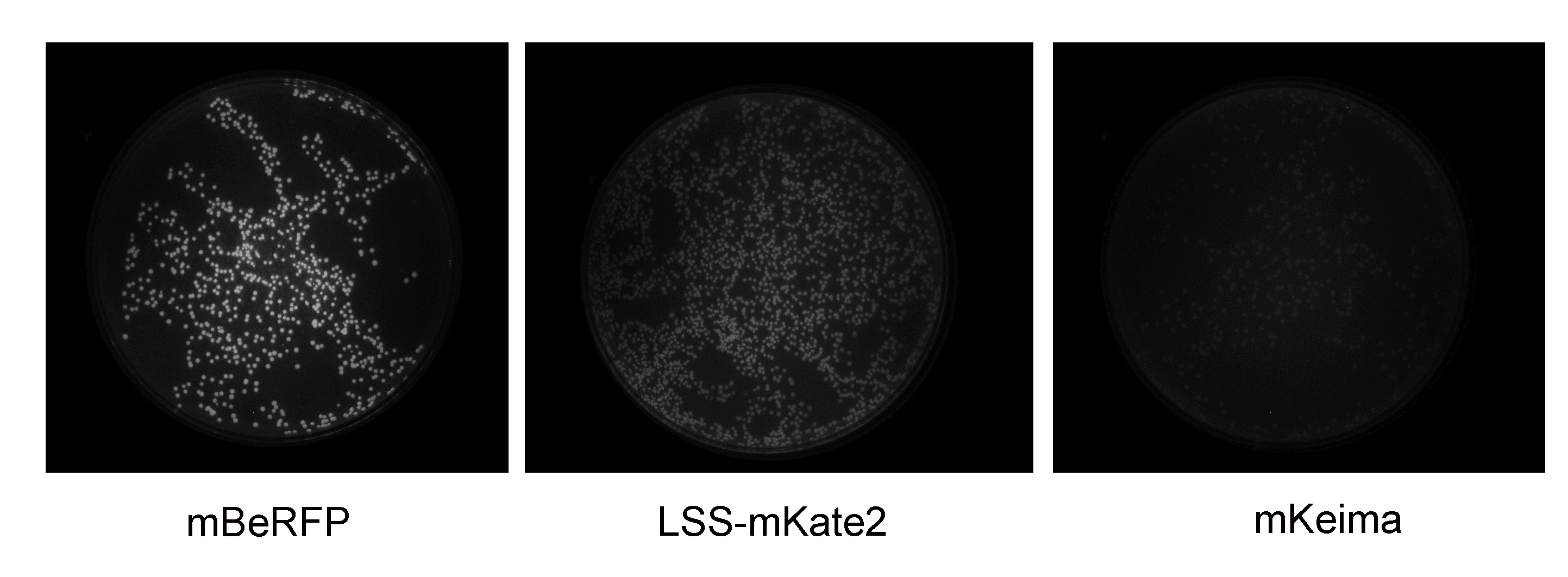

Supplement: Figure S3 — Brightness of mBeRFP, LSS-mKate2, and mKeima expressed in E. coli BL21(DE3) cells. The pRSET-mBeRFP, pRSET-LSS-mKate2, and pRSET-mKeima plasmids were transformed into E. coli BL21(DE3) cells, which were then incubated at 37°C for 24 hours. The images were acquired using a homemade imaging system with an excitation filter at 440–460 nm and an emission filter at 600–640 nm. (TIF) [file pone.0064849.s003.tif]
