# Supplementary material for: mBeRFP, an Improved Large Stokes Shift Red Fluorescent Protein
Source: PLoS One. 2013 Jun 20;8(6):e64849. doi: 10.1371/journal.pone.0064849 (PMC3688735; doi:10.1371/journal.pone.0064849)
Supplement: Table S1 — Oligonucleotide primers used in this report (mutated positions are underlined, and N indicates A, T, G, or C). (DOCX) [file pone.0064849.s004.docx]

**Supplementary table 1**

| V97S | 5’CCTGAGGGCTTCACATGGGAGAGAGTCACCACATACGAAGACGGGGGCG3’ |
| --- | --- |
| Y210S | 5’CAAGGAGGCCGACAAAGAGACCTACGTCGAGCAGCACGAGGTGGCTGTG3’ |
| L174X-F | 5’-GGGGGCCACCTGATCTGCAAC*NNN*AAGACCACATACAGATCCAAGAAACCCG-3’ |
| L174X-R | 5’CGGGTTTCTTGGATCTGTATGTGGTCTT*NNN*GTTGCAGATCAGGTGGCCCCC-3’ |
| S158X | 5'CACGAGCTTCAGGGCCATGTC*NNN*TCTGCCTTCCAGGCCGCCGT-3' |
| BAX-F | 5'AAAACTCGAGCTATGGACGGGT CCGGGGAGC3' |
| BAX-R | 5'AAAAGAATTCTCAGCCCATCTTCTTCCAGATGGTG3' |
| mBeRFP-F | 5'CCCGGGATCCACCGGTCGCCACCATGGTGTCTAAGGGCGAAGAGC3 |
| mBeRFP-R | 5'CGCGGCCGCTTCATTTGTGCCCCAGTTTGCTAG3 |
